# Complex genetic patterns in closely related colonizing invasive species

**DOI:** 10.1002/ece3.258

**Published:** 2012-07

**Authors:** Aibin Zhan, John A Darling, Dan G Bock, Anaïs Lacoursière-Roussel, Hugh J MacIsaac, Melania E Cristescu

**Affiliations:** 1 Great Lakes Institute for Environmental Research, University of Windsor401 Sunset Avenue, Windsor, Ontario N9B 3P4, Canada; 2 National Exposure Research Laboratory, US Environmental Protection Agency109 T. W. Alexander Drive, Durham, North Carolina 27711, USA; 3 Department of Biology, McGill University1205 Docteur Penfield, Montréal, Québec H3A 1B1, Canada

**Keywords:** *Ciona intestinalis*, genetic complexity, phylogeography, population genetics, selection/local adaptation, solitary ascidian

## Abstract

Anthropogenic activities frequently result in both rapidly changing environments and translocation of species from their native ranges (i.e., biological invasions). Empirical studies suggest that many factors associated with these changes can lead to complex genetic patterns, particularly among invasive populations. However, genetic complexities and factors responsible for them remain uncharacterized in many cases. Here, we explore these issues in the vase tunicate *Ciona intestinalis* (Ascidiacea: Enterogona: Cionidae), a model species complex, of which spA and spB are rapidly spreading worldwide. We intensively sampled 26 sites (*N* = 873) from both coasts of North America, and performed phylogenetic and population genetics analyses based on one mitochondrial fragment (cytochrome c oxidase subunit 3–NADH dehydrogenase subunit I, COX3-ND1) and eight nuclear microsatellites. Our analyses revealed extremely complex genetic patterns in both species on both coasts. We detected a contrasting pattern based on the mitochondrial marker: two major genetic groups in *C. intestinalis* spA on the west coast versus no significant geographic structure in *C. intestinalis* spB on the east coast. For both species, geo-graphically distant populations often showed high microsatellite-based genetic affinities whereas neighboring ones often did not. In addition, mitochondrial and nuclear markers provided largely inconsistent genetic patterns. Multiple factors, including random genetic drift associated with demographic changes, rapid selection due to strong local adaptation, and varying propensity for human-mediated propagule dispersal could be responsible for the observed genetic complexities.

## Introduction

Identifying factors responsible for microevolutionary processes over contemporary timescales is important for understanding how species evolve in response to rapidly changing environments associated with recent anthropogenic activities (Carroll 2008; Roux and Wieczorek 2008). Biological invasions provide an opportunity to answer fundamental questions regarding microevolution, especially those involving rapid range expansion or habitat transitions (Lee 2002; Suarez and Tsutsui 2008; Winkler et al. 2008; Bock et al. 2012). Processes associated with biological invasions, such as transfers of individuals to dramatically different environments and sudden changes in population demography, can lead to exceedingly rapid evolutionary changes (Reznick and Ghalambor 2001; Lee 2002; Prentis et al. 2008). However, understanding evolutionary processes that may accompany biological invasions can be challenging because of genetic complexities generated by biological invasions (Lee 2002). Despite recent progress on evolutionary aspects of biological invasions, the role and importance of the microevolutionary factors remain poorly understood (see reviews by Lee 2002; Prentis et al. 2008).

To better understand these evolutionary mechanisms, it is necessary to identify potential generalities and inconsistencies within and among multiple invasive species across different geographic scales, and then identify the evolutionary/ecological factors associated with common and unique genetic patterns. For aquatic species, many factors including life-history characteristics (e.g., sexual vs. asexual reproduction, planktotrophic vs. direct development, etc.), environmental heterogeneity (e.g., different temperature, salinity, etc.), anthropogenic activities (e.g., habitat fragmentation, pollution, etc.), coupled with varying propensity for human-mediated propagule dispersal, can lead to extremely complex genetic patterns (Lodge 1993; Kolar and Lodge 2001; Sakai et al. 2001; Lee and Boulding 2009; Dupont et al. 2010; Bock et al. 2011). Recent empirical studies have revealed complex genetic patterns at different geographic scales in aquatic invasive species (see e.g., Ben-Shlomo et al. 2006; Dupont et al. 2009; Tepolt et al. 2009; Bock et al. 2011). Given that numerous nonindependent factors may be involved in determining genetic patterns of aquatic invasive species, the choice of an appropriate model system seems critically important.

The vase tunicate, *Ciona intestinalis*, is a well-known model species for developmental and evolutionary biology (see review by Procaccini et al. 2011). The recently published whole genome (Dehal et al. 2002), coupled with detailed phylo-genetic and population genetics analyses (Caputi et al. 2007; Sordino et al. 2008; Nydam and Harrison 2010; Zhan et al. 2010), has expanded *C. intestinalis* as a model for evolutionary studies associated with biological invasions. Phylogenetic analyses revealed that *C. intestinalis* is a species complex consisting of at least four morphologically cryptic but genetically distinct species (spA, spB, spC, spD; Zhan et al. 2010). Interestingly, the two highly invasive species, spA and spB, inhabit largely disjoint geographic regions worldwide, while the other two, spC and spD, remain restricted to their putative native ranges in the Mediterranean Sea and Black Sea, respectively (Caputi et al. 2007; Nydam and Harrison 2007; Zhan et al. 2010). Population genetics analyses further illustrated relatively low population differentiation and high population connectivity for the two highly invasive species at both regional and continental scales (Zhan et al. 2010). Abundant genetic information generated by intensive developmental and evolutionary studies, as well as increasing knowledge regarding phylogenetics and population biology, indicates that the *C. intestinalis* species complex is a promising system for comparing genetic patterns in closely related invasive species during range expansions.

**Figure 1:**
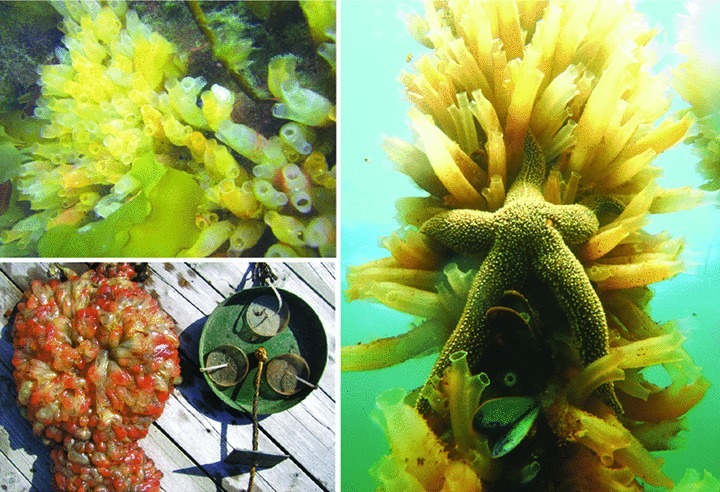
The vase tunicate, *Ciona intestinalis*, fouled with high density on hard substratum in the Port of Point Tupper (left upper), on sampling plates (before and after sampling, left lower), and on ropes for mussel aquaculture in Prince Edward Island (right). Photos by Anaïs Lacoursière-Roussel and Samuel Collin.

During the past century, the two highly invasive species of *C. intestinalis* complex, spA and spB, have successfully invaded coastal marine habitats throughout the temperate zone (Kott 1952; Monniot and Monniot 1994; Lambert and Lambert 2003; McDonald 2004). In invaded areas, these two species rapidly cover available substratum (Fig. 1) and exclude native species, often causing considerable economic damage and ecological changes (Lambert and Lambert 2003; Ramsay et al. 2008). In North America, *C. intestinalis* colonized the west coast either in the early 1910s (Huntsman 1912) or in the 1930s (Lambert and Lambert 1998). Uncertainty in its invasion history is largely due to the lack of distinct morphological attributes between *C. intestinalis* and its congener, *C. savignyi* (Lambert 2003). Owing to the absence of records on the east coast, the invasion history remains uncertain along almost all of this coast, with the exception of bays in Prince Edward Island, which were newly invaded in 2004 (Ramsay et al. 2008). So far, *C. intestinalis* has successfully colonized the Pacific coast of North America from British Columbia, Canada, to southern California, USA, and the Atlantic coast from Newfoundland, Canada, to Florida, USA (Therriault and Herborg 2008; de Oliveira Marins et al. 2009).

Here, we use phylogenetic and population genetics approaches based on mitochondrial DNA (mtDNA) sequences and nuclear microsatellites to characterize genetic patterns of 26 populations of *C. intestinalis* sampled from both coasts of North America. We aim to clarify population genetic structure of the two highly invasive species, *C. intestinalis* spA and spB on both coasts of North America. Based on these analyses, we discuss the potential factors driving complex genetic patterns, especially among closely related invasive species.

## Materials and Methods

### Sampling and species identification

**Figure 2:**
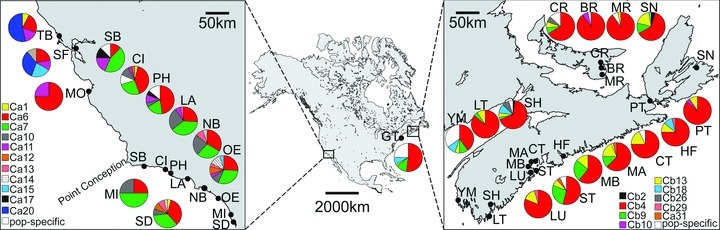
Sampling sites and distribution of mitochondrial cytochrome c oxidase subunit 3–NADH dehydrogenase subunit 1 (COX3-ND1) haplotypes for *Ciona intestinalis* spA on the west coast and spB on the east coast of North America. Site IDs as per Table 1. Pie charts indicate the proportion of haplotype groups observed at each site, with different colors corresponding to different haplotypes.

*Ciona intestinalis* specimens were collected from the invaded ranges on both coasts of North America. On the west coast, sampling was conducted along the coast of California between July and November 2006, spanning from Tomales Bay to San Diego estuary, while on the east coast sampling was performed between October 2007 and March 2009 in Prince Edward Island, Nova Scotia, and Connecticut (Fig. 2). In total, we sampled 26 populations (*N* = 873; Fig. 2; Table 1), including 11 from the west coast (*N* = 380) and 15 (*N* = 493; including populations BR, MR, PT, CT, LT, and GT from Zhan et al. 2010) from the east coast. All specimens were preserved in 95% ethanol prior to genetic analyses.

**Table 1:** Collection sites and genetic diversity for mitochondrial (mtDNA) cytochrome c oxidase subunit 3–NADH dehydrogenase subunit 1 (COX3-ND1) and microsatellites in two highly invasive species *Ciona intestinalis* spA (west coast) and spB (east coast) in North America. The first letter of the haplotype name denotes mtDNA (COX3-ND1) and the second letter indicates species affiliation: a, spA; b, spB. *N*, number of individuals tested; *n*, number of haplotypes; *h*, haplotypic diversity; π, nucleotide diversity; *A*, mean number of alleles; *A*_r_, mean allelic richness; *H*_O_, mean observed heterozygosity; *H*_E_, mean expected heterozygosity. The major ports are ranked based on shipping and other purposes such as cruise and local delivery

Sampling location	Habitat type	ID	mtDNA					Microsatellite				
				
			*N*	*N*	Haplotype	*H*	π	*N*	*A*	*A*_r_	*H*_O_	*H*_E_
West coast (*C. intestinalis* spA)												
Tomales Bay, CA, USA	Semi-enclosed bay	TB	13	4	Ca1, Ca6, Ca11, Ca20	0.679	0.0034	26	6.5	5.3	0.302	0.565
San Francisco Estuary, CA, USA	Estuary, port (major)	SF	9	3	Ca11, Ca20, Ca21	0.861	0.0036	32	7.4	5.8	0.231	0.622
Monterey Bay, CA, USA	Open sea shore	MO	8	2	C6, Ca11	0.429	0.0015	20	3.6	3.4	0.124	0.699
Santa Barbara, CA, USA	Marina	SB	16	6	Ca6, Ca7, Ca11, Ca15, Ca22, Ca23	0.783	0.0019	30	8.8	6.2	0.399	0.619
Channel Islands, CA, USA	Semi-enclosed port	CI	27	8	Ca1, Ca6–Ca12	0.772	0.0026	22	4.9	4.1	0.253	0.610
Port Hueneme, CA, USA	Semi-enclosed port (major)	PH	21	8	Ca6, Ca7, Ca10, Ca11, Ca16–Ca19	0.752	0.0028	20	4.1	3.9	0.219	0.563
Los Angeles, CA, USA	Port (major)	LA	31	5	Ca6, Ca7, Ca10, Ca11, Ca13	0.710	0.0016	34	7.0	5.5	0.245	0.576
Newport Bay, CA, USA	Semi-enclosed estuary, port	NB	21	5	Ca6, Ca7, Ca10, Ca13, Ca14	0.719	0.0016	24	6.6	5.2	0.310	0.702
Oceanside Estuary, CA, USA	Estuary, marina	OE	23	8	Ca6, Ca7, Ca10–Ca15	0.802	0.0027	30	8.5	6.7	0.377	0.703
Mission Bay, CA, USA	Semi-enclosed bay	MI	8	3	Ca6, Ca7, Ca10	0.714	0.0015	25	7.4	5.7	0.345	0.691
San Diego, CA, USA	Semi-enclosed bay, port (major)	SD	26	8	Ca1, Ca6, Ca7, Ca10, Ca12–Ca14	0.714	0.0022	38	10.4	7.0	0.397	0.692
Sub-total (West coast)		11	203	20		0.721	0.0023	301	183	5.3	0.291	0.640
East coast (*C. intestinalis* spB)												
Cardigan River, PE, Canada	Estuary, aquaculture farm	CR	30	8	Cb4, Cb9, Cb13, Cb24–Cb28	0.556	0.0019	30	9.4	7.4	0.299	0.823
Brudenell River, PE, Canada	Estuary, aquaculture farm	BR	30	3	Cb4, Cb10, Cb11	0.191	0.0004	29	9.1	7.2	0.337	0.810
Murray River, PE, Canada	Estuary, aquaculture farm	MR	30	3	Cb4, Cb12, Cb13	0.191	0.0004	30	8.9	7.1	0.309	0.805
Sydney, NS, Canada	Port (major)	SN	42	5	Cb2, Cb4, Cb9, Cb13, Cb18	0.577	0.0020	23	8.5	7.2	0.268	0.812
Point Tupper, NS, Canada	Port (major)	PT	21	3	Cb4, Cb10, Cb13	0.267	0.0005	33	10.1	6.9	0.363	0.771
Halifax, NS, Canada	Port (major)	HF	28	4	Cb4, Cb13, Cb18, Cb29	0.429	0.0031	21	10.1	7.9	0.314	0.826
Chester, NS, Canada	Marina	CT	28	4	Cb4, Cb13–Cb15	0.418	0.0012	25	11.1	8.2	0.357	0.832
Martin's River, NS, Canada	Estuary	MA	46	6	Cb4, Cb9, Cb10, Cb13, Cb30, Cb31	0.592	0.0015	24	4.1	3.7	0.196	0.510
Mahone Bay, NS, Canada	Bay	MB	28	3	Cb4, Cb9, Cb13	0.569	0.0012	24	5.4	4.3	0.242	0.611
Stone Hurst, NS, Canada	Bay	ST	28	7	Cb4, Cb9, Cb13, Cb24, Cb31–Cb33	0.798	0.0019	20	5.9	5.0	0.201	0.653
Lunenburg, NS, Canada	Open sea shore	LU	21	3	Cb4, Cb13, Cb29	0.338	0.0008	31	6.8	5.3	0.236	0.674
Shelburne, NS, Canada	Port	SH	39	7	Cb2, Cb4, Cb9, Cb13, Cb18, Cb26, Cb34	0.547	0.0039	25	8.6	6.6	0.279	0.755
Port La Tour, NS, Canada	Marina	LT	21	3	Cb4, Cb9, Cb13	0.292	0.0006	21	8.6	6.8	0.297	0.794
Yarmouth, NS, Canada	Port	YM	20	8	Cb4, Cb9, Cb13, Cb18, Cb35–Cb38	0.821	0.0062	24	12.6	9.3	0.355	0.875
Groton, CT, USA	Estuary, port	GT	48	6	Cb4, Cb9, Cb13, Cb17–Cb19	0.688	0.0043	48	15.0	8.9	0.416	0.849
Sub-total (East coast)		15	460	27		0.485	0.0020	408	239	6.8	0.298	0.760
Total		26	663	46		0.603	0.0022	709	422	6.1	0.295	0.700

Owing to the lack of reliable diagnostic morpho-logical characters among species in *C. intestinalis* complex, all collected specimens were identified to species level using one mtDNA fragment, cytochrome c oxidase subunit 3–NADH dehydrogenase subunit 1 (COX3-ND1; Zhan et al. 2010).

### mtDNA amplification and sequencing

Total genomic DNA was extracted from approximately 50 mg of tissue according to the proteinase *K* method (Waters et al. 2000). The mitochondrial COX3-ND1 fragment was amplified using the primers TX3F and TN1R (Iannelli et al. 2007). PCR amplification was performed according to the protocol described by Iannelli et al. (2007). Sequencing reactions were performed using the forward primer (TX3F), BigDye Terminator v3.1 sequencing chemistry, and an ABI 3130XL automated sequencer (PE Applied Biosystems, Foster City, CA). Sequences that contained ambiguous nucleotides were subsequently sequenced using the reverse primer (TN1R).

### mtDNA analysis

Mitochondrial sequences were aligned and edited using CodonCode Aligner v2.0.6 (CodonCode Corporation, Dedham, MA). Bayesian inference (BI) and neighbor-joining (NJ) phylogenetic analyses were conducted using *C. savignyi* as outgroup (Nydam and Harrison 2007; Zhan et al. 2010). The best DNA substitution model for mtDNA haplotypes was determined using MODELTEST v3.7 (Posada and Crandall 1998) with Akaike Information Criterion. The BI analysis was conducted using MRBAYES v3.2 (Ronquist and Huelsenbeck 2003). Trees were sampled every 100 generations for two million generations, and the first 25% of all the trees sampled were discarded as burn-in. NJ phylo-genetic analyses were performed using MEGA v4.0 (Tamura et al. 2007) based on nucleotide distances corrected using the Tamura–Nei model (Tamura and Nei 1993). Clade support was estimated using bootstrap analysis with 1000 replicates. Relationships between mtDNA haplotypes were further examined using a statistical parsimony haplotype network generated at the 95% connection limit with TCS v1.21 (Clement et al. 2000).

The number of haplotypes (*n*), haplotype diversity (*h*), and nucleotide diversity (π) were assessed using DNASP v5 (Rozas et al. 2003). Population pairwise Φ_ST_ was calculated using the Tamura and Nei (TrN) substitution model and 10,000 permutations in ARLEQUIN v3.1 (Excoffier et al. 2005), with levels of significance adjusted using sequential Bonferroni corrections (Rice 1989). To assess hierarchical population genetic structure, we conducted a hierarchical analysis of molecular variance (AMOVA; Excoffier et al. 1992) based on 10,000 random permutations using ARLEQUIN. AMOVA was performed separately for both coasts (i.e., two species, spA on the west coast and spB on the east coast, see Results section). Populations were grouped according to different geographical regions on both coasts, two groups on the west coast: northern California (TB, SF, MO) and southern California (SB, CI, PH, LA, NB, OE, MI, SD; Table 1, Fig. 2); three groups on the east coast: Prince Edward Island (CR, BR, MR), Nova Scotia (SN, PT, HF, CT, MA, MB, ST, LU, SH, LT, YM), and Connecticut (GT; Table 1; Fig. 2). Molecular variance was partitioned into three levels: between regions, among populations within regions, and within populations. To test the correlation between genetic distance [Φ_ST_/(1–Φ_ST_)] and geographic distance, we performed isolation by distance (IBD) analysis for spA and spB using a Mantel test with 10,000 permutations implemented in GENEPOP v3.4 (Raymond and Rousset 1995). Geographical distances were calculated as the minimum coastline distances between sampling locations using Google Earth.

### Microsatellite DNA genotyping and analysis

All individuals were genotyped for eight polymorphic microsatellite markers (Zhan et al. 2010). PCR amplification and genotyping were performed according to the protocol described by Zhan et al. (2010). Microsatellite genetic diversity was evaluated by the number of alleles (*A*), allelic frequency (*F*), allelic richness (*A*_r_), as well as the observed heterozygosity (*H*_O_) and expected heterozygosity (*H*_E_) using F_STAT_ v2.9.3.2 (Goudet 2001). Additionally, a nonparametric test (Mann–Whitney *U* test), implemented in STATISTICA v6 (StatSoft, Inc., Tulsa, OK), was used to test the difference in allelic richness (*A*_r_) and expected heterozygosity (*H*_E_) among populations. Markov chain method (Guo and Thompson 1992) was employed to estimate the probability of significant deviation from Hardy–Weinberg equilibrium (HWE) using GENEPOP. Significance criteria were adjusted for the number of simultaneous tests using sequential Bonferroni corrections.

Population differentiation was determined by *F*_ST_ (Weir and Cockerham 1984) for all population pairs using F_STAT_. A total of 10,000 permutations were performed, and significance levels were adjusted using sequential Bonferroni corrections. In addition, population structure was determined by conducting a three-dimensional factorial correspondence analysis (3D-FCA) in GENETIX v4.05 (Belkhir et al. 2004) and a Bayesian, Markov Chain Monte Carlo (MCMC) based approach implemented in STRUCTURE v2.1 (Pritchard et al. 2000). For the STRUCTURE analysis, we assessed likelihoods for models with the number of clusters ranging from *K* = 1 to the total number of sampling sites (11 for spA and 15 for spB). Ten independent runs were performed for each specified *K*-value, and for each run, 1,000,000 generations were used after discarding 100,000 generations as burn-in. The measure of Δ*K* (Evanno et al. 2005) was used to infer the number of biologically relevant clusters. To test for a pattern of IBD, we used a Mantel procedure with 10,000 permutations implemented in GENEPOP to assess the dependence between the genetic distances [*F*_ST_/(1–*F*_ST_)] and geographic distances in each species. A hierarchical AMOVA was performed in ARLEQUIN based on microsatellite genetic distances between populations, and partitioning variance as for mtDNA.

## Results

### mtDNA analyses

**Figure 3:**
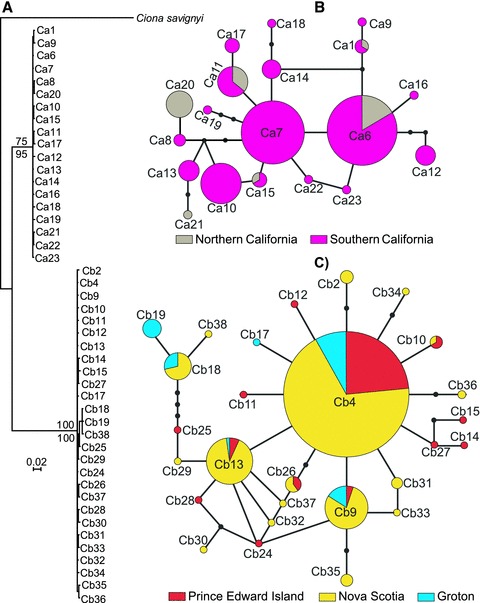
Bayesian inference (BI) tree (A) based on mitochondrial cytochrome c oxidase subunit 3–NADH dehydrogenase subunit 1 (COX3-ND1) haplotypes, and haplotype networks generated with TCS for *Ciona intestinalis* spA (B) on the west coast and *C. intestinalis* spB (C) on the east coast of North America. Haplotype and population names as per Table 1. For the BI tree, posterior probabilities for Bayesian inferences (in percentage, above branch) and bootstrap values (below branch) for neighbor-joining reconstruction are shown at major nodes. For the TCS network, sampled haplotypes are indicated by circles and missing or unsampled haplotypes are indicated by solid black dots. Haplotypes are color coded according to different geographical regions.

A total of 663 individuals derived from 26 populations from both coasts were successfully sequenced for the COX3-ND1 fragment (Table 1). Species identification based on phylo-genetic analysis of this fragment recovered two well-supported phylogroups, corresponding to spA and spB (Fig. 3A). The mean interclade divergence was 15.9%, much higher than the intraclade divergences (spA: 0.5%; spB: 0.8%). All individuals from west coast were identified as spA, while all individuals from east coast corresponded to spB (Table 1; Fig. 3A).

In total, we identified 19 haplotypes (GenBank accession numbers: HM036361, HM036366, JQ396394–JQ396310) for spA and 27 haplotypes (GenBank accession numbers: HM036368, HM036370, HM036375–HM036381, HM036383–HM0385, JQ396311–JQ396325) for spB (Table 1; Fig. 2). The number of haplotypes per population ranged from two to eight for spA and three to eight for spB, while the haplotype diversity (*h*) varied from 0.429 to 0.861 for spA, and 0.191 to 0.821 for spB (Table 1). The lowest genetic diversity (*h* = 0.429) in spA was found in the population MO (Monterey Bay), sampled from the open sea shore on the west coast, while the highest was detected in major ports including SF (San Francisco, *h* = 0.861), PH (Port Hueneme, *h* = 0.752), and SD (San Diego, *h =* 0.714; Table 1). Similarly, some populations of spB sampled from ports showed relatively high genetic diversity (e.g., *h* = 0.577 for population Sydney; Table 1), but some did not (e.g., *h* = 0.267 for population Point Tupper; Table 1). Two of the most recently established populations, BR (Brudenell River; reported in 2005, C. D. Mills, personal communication) and MR (Murray River; detected in 2006, C. D. Mills, personal communication), sampled from aquaculture facilities in Prince Edward Island, exhibited the lowest haplotype diversity (*h* = 0.191; Table 1). By contrast, one recently established neighboring population CR (Cardigan River; reported in 2006, C. D. Mills, personal communication) showed a similar level of genetic diversity (*h =* 0.556) to that in other populations (Table 1).

The shallow phylogeny within each species was confirmed by TCS haplotype networks. Haplotypes were connected to each other by only several mutation steps (Fig. 3B and C). The network for spB showed a star-shaped pattern with one dominant haplotype at the center (Fig. 3C), whereas we detected two dominant haplotypes at the center for spA (Fig. 3B). Despite the shallow phylogeny and close relationships among haplotypes, we detected geographic structure for spA along the west coast. Generally, populations located at two sides of Point Conception belonged to two geographic groups, that is, southern and northern California (Fig. 2). The haplotype composition and haplotype frequencies were different in these two geographic groups (Table 1; Fig. 2). For example, the most dominant haplotype in southern California, Ca7, was not detected in any locations in northern California. Similarly, haplotypes with relatively high frequencies in northern California, such as Ca20, were not observed in southern California (Fig. 2). The difference between two groups was further confirmed by both pairwise Φ_ST_ values (Table 2A) and AMOVA (Table 3). Comparisons between populations sampled from northern and southern California revealed high and significant Φ_ST_ values in more than half of the population pairs (14 out of 27; Table 2A), while low and nonsignificant values were detected for population pairs within each region (Table 2A). AMOVA attributed a statistically significant proportion of genetic variance (14.53%, *P* < 0.005) to the among-group component (i.e., between two groups, Table 3).

**Table 2:** Estimates of population genetic differentiation in two highly invasive vase tunicates: *Ciona intestinalis* spA on the west coast (A) and spB on the east coast (B) of North America. Above diagonal: pairwise Ф_ST_ based on cytochrome c oxidase subunit 3–NADH dehydrogenase subunit 1 region (COX3-ND1); Below diagonal: pairwise *F*_ST_ based on microsatellite markers. Bold numbers indicate statistical significance after sequential Bonferroni corrections. Negative values were converted into zero

(A)	TB	SF	MO	SB	CI	PH	LA	NB	OE	MI	SD
TB	****	0.000	0.111	**0.367**	**0.255**	**0.336**	**0.339**	**0.299**	**0.311**	**0.265**	**0.318**
SF	0.022	****	0.027	**0.185**	**0.094**	0.174	**0.119**	**0.092**	0.129	0.043	**0.144**
MO	**0.092**	**0.085**	****	**0.264**	0.019	0.081	0.198	0.142	0.120	0.143	0.081
SB	**0.063**	**0.060**	**0.069**	****	0.120	0.061	0.120	0.147	0.069	0.100	0.114
CI	0.022	0.013	**0.105**	0.049	****	0.008	0.032	0.000	0.000	0.000	0.000
PH	**0.095**	**0.087**	**0.074**	**0.079**	**0.123**	****	0.082	0.068	0.011	0.033	0.007
LA	0.024	0.000	**0.087**	0.033	0.048	**0.089**	****	0.000	0.018	0.000	0.036
NB	0.000	0.003	**0.082**	**0.057**	0.036	**0.080**	0.000	****	0.005	0.000	0.006
OE	**0.092**	**0.078**	**0.097**	**0.050**	**0.111**	**0.079**	**0.077**	**0.051**	****	0.000	0.000
MI	0.021	0.007	**0.098**	**0.059**	0.035	**0.095**	0.011	0.013	**0.081**	****	0.000
SD	**0.077**	**0.069**	**0.097**	0.015	**0.078**	**0.082**	**0.056**	**0.053**	0.037	**0.068**	****

(B)	CR	BR	MR	SN	PT	HF	CT	MA	MB	ST	LU	SH	LT	YM	GT
CR	****	0.058	0.041	0.000	0.003	0.035	0.000	0.000	0.010	0.062	0.000	0.012	0.000	0.125	0.104
BR	0.019	****	0.023	0.073	0.006	0.115	0.071	0.092	**0.153**	**0.233**	0.102	0.056	0.033	**0.236**	**0.153**
MR	0.005	0.015	****	0.054	0.006	0.100	0.046	0.076	0.136	**0.220**	0.065	0.048	0.006	**0.229**	**0.147**
SN	**0.057**	**0.059**	**0.057**	****	0.013	0.024	0.007	0.000	0.005	0.056	0.000	0.019	0.000	0.131	0.099
PT	**0.044**	0.030	**0.038**	0.033	****	0.054	0.000	0.026	0.088	0.168	0.000	0.023	0.000	0.176	0.116
HF	0.034	0.026	0.029	**0.051**	0.039	****	0.043	*0.065*	0.090	0.130	0.021	0.000	0.054	0.054	0.020
CT	0.030	0.018	0.035	**0.066**	0.035	0.037	****	0.021	0.071	0.144	0.000	0.030	0.000	0.175	0.120
MA	**0.162**	**0.175**	**0.174**	**0.176**	**0.148**	**0.152**	**0.125**	****	0.000	0.039	0.010	0.049	0.007	0.169	**0.132**
MB	**0.168**	**0.182**	**0.174**	**0.180**	**0.186**	**0.166**	**0.156**	0.009	****	0.000	0.075	0.056	0.045	0.139	0.120
ST	**0.147**	**0.169**	**0.163**	**0.129**	**0.154**	**0.152**	**0.124**	**0.150**	**0.097**	****	0.148	0.094	0.129	0.123	0.131
LU	**0.111**	**0.125**	**0.115**	**0.130**	**0.129**	**0.123**	**0.111**	0.016	0.031	**0.074**	****	0.014	0.000	0.145	0.100
SH	**0.054**	0.043	**0.047**	**0.047**	0.009	0.027	0.030	**0.174**	**0.191**	**0.155**	**0.131**	****	0.022	0.062	0.037
LT	**0.047**	0.030	0.036	**0.068**	0.036	0.039	0.033	**0.154**	**0.185**	**0.179**	**0.146**	0.032	****	0.164	0.112
YM	**0.048**	**0.037**	**0.054**	0.042	**0.062**	0.035	**0.048**	**0.153**	**0.168**	**0.147**	**0.134**	**0.054**	**0.055**	****	0.023
GT	**0.036**	**0.030**	**0.039**	**0.067**	**0.053**	**0.046**	0.005	**0.124**	**0.152**	**0.111**	**0.120**	**0.048**	**0.043**	**0.053**	****

**Table 3:** Results of the analysis of molecular variance (AMOVA) for two highly invasive species, *Ciona intestinalis* spA on the west coast and spB on the east coast of North America. Populations were grouped according to different geographic regions on both coasts, two groups on the west coast: northern California (TB, SF, MO) and southern California (SB, CI, PH, LA, NB, OE, MI, SD); three groups on the east coast: Prince Edward Island (CR, BR, MR), Nova Scotia (SN, PT, HF, CT, MA, MB, ST, LU, SH, LT, YM), and Connecticut (GT)

Grouping	Source of variation	Sum of square	Variance components	Percentage variation	*P* value
spA	(A) mtDNA				
West coast	Among groups	3.78	0.06	14.53	0.00
	Among populations within groups	4.36	0.01	1.27	0.14
	Among individuals within populations	72.58	0.38	84.21	0.01
	Total	80.73	0.45		
	(B) Microsatellite				
	Among groups	9.56	–0.01	–0.21	0.00
	Among populations within groups	92.50	0.19	7.18	0.00
	Among individuals within populations	1132.72	2.42	93.04	0.54
	Total	1234.77	2.60		
spB	(C) mtDNA				
East coast	Among groups	3.09	0.01	3.42	0.00
	Among populations within groups	6.04	0.01	3.32	0.00
	Among individuals within populations	109.66	0.25	93.26	0.03
	Total	118.79	0.27		
	(D) Microsatellite				
	Among groups	55.63	0.02	0.55	0.00
	Among populations within groups	213.63	0.33	9.65	0.00
	Among individuals within populations	2206.34	3.08	89.79	0.22
	Total	2475.60	3.43		

By contrast, we did not observe significant geographic structure at the mtDNA locus for spB along the east coast. Generally, all populations shared the same dominant haplotype, Cb4 (Table 1; Fig. 2). Statistical analysis of pairwise Φ_ST_ values revealed that only eight of 120 pairs were significantly different after sequential Bonferroni correction (Table 2B). AMOVA attributed only a small proportion of genetic variance (3.42%) to the among-group component, although it was statistically significant (Table 3).

**Figure 4:**
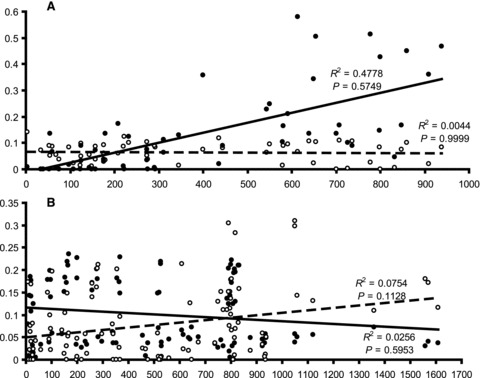
Correlation between geographical distance in kilometers (*x*-axis) and genetic distance given as *F*_ST_/(1 –*F*_ST_) for microsatellite data and Φ_ST_/(1 –Φ_ST_) for mitochondrial data (*y*-axis) in *Ciona intestinalis* spA on the west coast (A) and spB on the east coast (B) of North America. Open and solid cycles represent data derived from mitochondrial and microsatellite markers, respectively.

Mantel tests based on the COX3-ND1 fragment revealed no correlation between genetic and geographical distances for either species on both coasts (Fig. 4), suggesting no pattern of IBD.

### Microsatellite DNA analyses

A total of 709 individuals from 26 populations, including 301 individuals from 11 populations for spA and 408 individuals from 15 populations for spB, were successfully genotyped at eight microsatellite loci. In total, we identified 183 and 239 alleles for spA and spB, respectively. Allelic richness (*A*_r_) ranged from 3.4 to 7.0 (mean 5.3) for spA and from 3.7 to 9.3 (mean 6.8) for spB (Table 1). Generally, high expected hetero-zygosity (*H*_E_) values were observed for all populations, ranging from 0.563 to 0.703 for spA and from 0.510 to 0.875 for spB (Table 1; Table S1). For spA, the population MO (Monterey Bay) had the lowest genetic diversity, all consistent with mtDNA results (Table 1). Allelic richness in this population was significantly lower than three populations (LA, OE, SD) (Mann–Whitney *U* test, *P* < 0.013; Table S2). We did not detect statistical difference in expected hetero-zygosity between any population pairs (*P* > 0.05; Table S2). Similarly to mtDNA, populations sampled from major ports showed relatively high genetic diversity (Table 1).

Interestingly, nuclear and mtDNA markers often provided inconsistent results. For example, for spA population PH (Port Hueneme) had the highest number of mtDNA haplotypes (*n* = 8; Table 1) but a relatively low number of alleles at microsatellite loci (mean *A* = 4.1; Table 1). For spB, the lowest microsatellite genetic variation was identified in four populations including MA (Martin's River), MB (Mahone Bay), ST (Stone Hurst), and LU (Lunenburg), which were sampled from southeastern Nova Scotia (Tables 1 and S1). Both allelic richness and expected heterozygosity were significantly lower than those in other populations (Mann–Whitney *U* test; Table S2). However, two of these four populations, MA and ST, exhibited high mitochondrial diversity (Table 1). Compared to the other populations, the populations sampled from major ports such as HF (Halifax) did not show higher genetic diversity, and recently established populations in Prince Edward Island did not exhibit significantly lower genetic diversity (Table 1). Deviations from HWE were observed at multiple loci and sampling locations for both species (Table S1). All of the deviant cases showed significant heterozygote deficiency (*P* < 0.001; Table S1). As suggested by Zhan et al. (2010), recurrent inbreeding and Wahlund effects may be responsible for these deviations.

Overall, we detected a relatively high level of genetic differentiation in both species when analyzing microsatellite data. For spA, pairwise *F*_ST_ values ranged from zero to 0.123 with an average of 0.059. In total, 36 of 55 (65.5%) population pairs were significantly different after Bonferroni correction (Table 2A). Interestingly, the highest genetic differentiation (*F*_ST_ = 0.123) was detected between two neighboring populations, CI (Channel Islands) and PH (Port Hueneme; Fig. 2; Table 2A). For spB, pairwise *F*_ST_ values varied from 0.005 to 0.191, with an average of 0.088. In total, 75 of 105 (71.4%) comparisons of population pairs remained significantly different after Bonferroni correction (Table 2B). The highest *F*_ST_ values were detected for comparisons between four populations (MA, MB, ST, LU) and all other sites surveyed, with an average of pairwise *F*_ST_ values greater than 0.1 (Table 2B).

**Figure 5:**
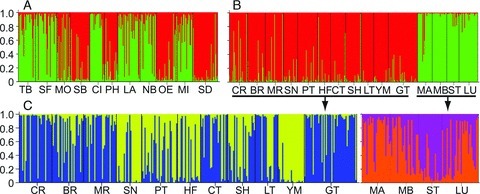
Bayesian inference (BI) of population structure for *Ciona intestinalis* spA (A), spB (B), and data subset of spB (C) based on microsatellite markers. For Bayesian clustering analysis, each genotype is represented by a thin vertical line, with proportional membership in different clusters indicated by colors. Bold vertical lines separate collection sites, with site IDs indicated below the plot.

**Figure 6:**
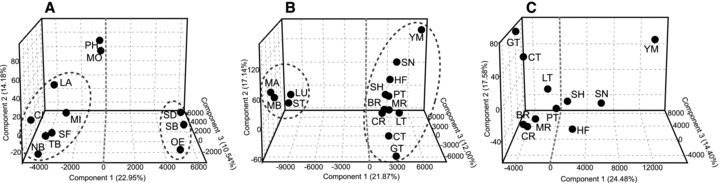
Three-dimensional factorial correspondence analysis (3D-FCA) of *Ciona intestinalis* spA (A), spB (B), and data subset of spB (C) after removing four populations (MA, MB, ST, and LU) based on eight polymorphic microsatellites.

The two different approaches used to infer population structure, 3D-FCA and Bayesian clustering, provided largely consistent results in both species. For spA on the west coast, both analyses grouped populations into two clusters based on geographic regions (i.e., northern and southern California), which is inconsistent with mtDNA results obtained from pairwise Φ_ST_ (Table 2A) and AMOVA (Table 3). Bayesian clustering analysis suggests a two-cluster model (*K* = 2) as the most parsimonious possibility (Fig. 5A). Most individuals from five populations (TB, SF, CI, LA, MI) were assigned into one cluster regardless of geographic origin, whereas most individuals from three populations (SD, SB, OE) collected from southern California were assigned to the other cluster (Fig. 5A). However, individuals from the remaining two populations, PH (Port Hueneme) and MO (Monterey Bay), do not appear to assign consistently to a single cluster (Fig. 5A). This pattern was confirmed by 3D-FCA. Component 1, explaining 22.95% of genetic variance, separated the five (TB, SF, CI, LA, MI) from the three populations (SD, SB, OE), with the remaining two (PH, MO) in the middle (Fig. 6A). For spB on the east coast, the two analyses consistently supported a two-cluster model and clearly separated four populations (MA, MB, ST, LU; Figs. 5B and 6B), all consistent with microsatellite diversity results (Table 1) and pairwise *F*_ST_ values (Table 2B). Since low genetic diversity in these four populations can inflate genetic differentiation, 3D-FCA and Bayesian clustering were rerun after removing these four populations. Bayesian clustering analysis still supported a two-cluster model, showing a high level of admixture and mixture of individuals from different geographical origins (Fig. 5C). This finding is consistent with the 3D-FCA results, showing no obvious population cluster (Fig. 6C). Similar to spA, the two clusters showed discontinuous distributions along the east coast. Neighboring populations were not assigned to a single cluster (e.g., CT and SH), whereas geographically isolated populations were frequently assigned to the same clusters (e.g., GT and CR). In addition, Mantel tests based on microsatellite data showed no indication of IBD in both species (Fig. 4).

## Discussion

Our genetic surveys based on both population genetics and phylogenetic analyses show complex genetic patterns in two closely related invasive species, *C. intestinalis* spA and spB. A contrasting geographic pattern was detected in the two species based on the mitochondrial marker: two major genetic groups in spA on the west coast versus no significant geographic structure in spB on the east coast (Fig. 2; Table 2). For both species on both coasts, multiple analyses showed that geographically distant populations were often genetically similar, whereas neighboring populations were sometimes not (Figs. 5 and 6). In addition, we observed inconsistent genetic patterns when using mitochondrial versus nuclear markers. All these genetic complexities suggest that multiple factors, coupled with their interactions, could be responsible for the genetic patterns observed in *Ciona*.

### Geographic structure based on mtDNA in spA on the west coast

Genetic analyses based on mtDNA revealed contrasting patterns between spA on the west coast and spB on the east coast: two major geographic groups in spA versus no significant geographic structure in spB (Fig. 2). For spA on the west coast, the genetic patterns can result from three possible processes: (1) separate introductions from genetically distinct sources; (2) random genetic drift associated with population demography; and/or (3) rapid selection due to strong local adaptation.

Given extremely heavy shipping to the west coast of North America (see e.g., Sylvester et al. 2011), it is possible that separate introductions may have occurred in both *Ciona* and other ascidians (see e.g., Stoner et al. 2002; Zhan et al. 2010; Bock et al. 2011). However, analyses based on microsatellites suggest that this process is unlikely responsible for defining the two geographic groups. Two tests, including 3D-FCA and Bayesian clustering, did not separate northern from southern populations (Figs. 5A and 6A). In addition, microsatellite analyses such as pairwise *F*_ST_ values suggest a high level of gene flow between several northern and southern populations. For example, there is no detectable microsatellite-based genetic differentiation between populations TB (Tomales Bay) and NB (Newport Bay), located in two different geographic groups as defined by mtDNA (Fig. 2; Table 2). Additional evidence for supporting this argument comes from little genetic structure based on global sampling and population genetics and phylogeographic analyses using both nuclear and mtDNA markers (Zhan et al. 2010). Altogether, available evidence suggests that separate introductions are unlikely responsible for such a genetic pattern in spA on the west coast.

Genetic drift can create complex genetic patterns by randomly changing allele frequencies and/or eliminating low-frequency alleles, especially when effective population sizes (*N*_e_) decrease. Moreover, compared to nuclear markers, mtDNA has smaller effective population size (*N*_e mtDNA_≍ 1/4 *N*_e nuclear_), likely leading to stronger signatures of genetic drift on mtDNA. For broadcast spawning marine invasive species including *Ciona*, two possible processes—sweepstakes reproductive success and dramatic demographic changes during translocation—can lead to strong genetic differentiation between populations. Marine species with broadcast spawning exhibit sweepstakes reproductive success, in which a subset of the population contributes the majority of offspring to subsequent generations (Hedgecock et al. 2007). This can sharply decrease effective population size, resulting in spatial and/or temporary genetic structuring (see e.g., Li and Hedgecock 1998, Hedgecock et al. 2007; Christie et al. 2010). The existence of population-specific haplotypes/alleles (Fig. 2; Table S1) suggests that sweepstakes reproductive success might occur in *Ciona*. In addition, population bottlenecks may have occurred during the translocation and settlement on the west coast. Random genetic drift due to severe bottlenecks, especially for mtDNA that is more sensitive to effective population size changes, may have created divergent genetic groups at initial stages of invasions when limited numbers of propagules were transplanted, and then these divergent genetic groups were transported to other locations *via* secondary spread.

However, genetic drift alone seems inadequate to explain the genetic pattern on the west coast, given the inconsistency between unique dominant haplotypes to specific geographic regions (Table 1; Fig. 2) and high gene flow between these regions suggested by microsatellite analyses (Table 2A; Figs. 5A and 6A). This mitochondrial-nuclear discrepancy (i.e., high mtDNA differentiation vs. no or very low genetic differentiation at nuclear loci) has been detected in several other marine species including those with relatively high self-dispersal capacity (e.g., a marine goby *Pomatoschistus minutus*; Larmuseau et al. 2010). Selection and sex-biased dispersal are generally adopted as major explanations for this discrepancy (see Larmuseau et al. 2010 and references therein). However, given that *Ciona* is hermaphroditic, sex-biased dispersal cannot be a valid explanation. Considering a high level of genetic exchange among populations suggested by micro-satellite analyses (Table 2A; Figs. 5A and 6A) and possible population connection mediated by frequent shipping, such differences at mtDNA between northern and southern California may be maintained by rapid selection associated with strong local adaptation. The two geographic groups detected on the west coast are located on two sides of a well-known oceanographic and biogeographic boundary, Point Conception (PC), where multiple species exhibit phylogeographic breaks (see e.g., Burton 1998; Bernardi 2000; Dawson et al. 2006; Earl et al. 2010). PC is marked by strong discontinuities in water temperature, salinity, dissolved oxygen, as well as hydrography (Burton 1998; Dawson et al. 2001). Strong upwelling and high wave exposure dominate the north of PC, while weak and seasonal upwelling and warmer water temperatures are characteristics in the south (Blanchette et al. 2007). These oceanographic conditions have important effects on benthic communities, influencing both growth rate and recruitment dynamics (see e.g., Connolly et al. 2001; Blanchette et al. 2007). Therefore, *Ciona* populations might locally adapt to these two dramatically distinct environments. Compared to neutral nuclear makers such as microsatellites, selection events are much easier to capture through mtDNA, mainly because the entire mitochondrial genome is one linkage group without recombination. A selective sweep due to any selection pressure on any mtDNA gene can easily fix the haplotypes having higher fitness. Indirect and direct selections on mtDNA have been demonstrated in many species (e.g., reviews by Ballard and Rand 2005; Hurst and Jiggins 2005), although genetic mechanisms for such selections on mtDNA remain poorly understood. Consequently, the observed pattern on the west coast in spA likely resulted from the interaction between random genetic drift associated with demographic change and rapid selection due to strong local adaptation.

Additional evidence suggests that the interaction between genetic drift and selection could be responsible for such a geographic pattern. When our data are combined with the global data from Zhan et al. (2010), we observed different dominant, and sometimes unique, haplotypes on different continents, although only several mutation steps were observed between these haplotypes (Fig. S1). This pattern was found not only in spA but also in spB. For instance, all haplotypes of spB in Asia are unique (Fig. S1).

### Complex intraspecific genetic patterns in both species

Multiple analyses show that intraspecific genetic patterns varied significantly across the sampling regions analyzed. High genetic differentiation (*F*_ST_ values) was observed not only between geographically distant populations, but also between adjacent ones (Table 2). IBD analyses did not reveal any significant correlation between genetic differentiation and geographical proximity for either species based on both types of markers (Fig. 4). Both Bayesian clustering and 3D-FCA occasionally assigned geographically distant populations into the same clusters (Figs. 5 and 6).

Recent empirical studies have revealed that human-mediated dispersal may leave strong signatures on population genetic structures in numerous invasive species, including ascidians (see e.g., Dupont et al. 2010; Zhan et al. 2010; Bock et al. 2011). Human-mediated gene flow can both generate complex genetic structuring at regional and local scales (see e.g., Darling and Folino-Rorem 2009; Dupont et al. 2010; Bock et al. 2011), and affect population genetic patterns among continents (see, e.g., Zhan et al. 2010). Generally, aquaculture transfer and shipping are considered as the leading pathways for human-mediated dispersal of ascidians in North America (Dijkstra et al. 2007; Zhan et al. 2010; Bock et al. 2011). Our genetic analyses detected apparent signatures of these two pathways on population genetic structure in *Ciona*. The major pathway responsible for introductions of *Ciona* to Prince Edward Island is aquaculture trade between Prince Edward Island and Nova Scotia (Dijkstra et al. 2007). Our genetic analysis is concordant with this view. For example, the population CT (Chester), sampled from Chester basin supporting aquaculture in Nova Scotia, showed no significant genetic differentiation from the three populations (CR, BR, MR) sampled from aquaculture facilities in Prince Edward Island (Table 2). Genetic analyses, including pairwise *F*_ST_, 3D-FCA, and Bayesian clustering, suggest long-distance gene flow between geographically distant populations in both species on both coasts (Table 2; Figs. 5 and 6), all consistent with population connectivity via watercraft-mediated transportation of propagules. The new evidence found here, coupled with that from Zhan et al. (2010), suggests that intraspecific genetic complexities largely resulted from genetic exchanges assisted by anthropogenic transfers, possibly from transportation of large numbers of larvae inside ballast tanks, and/or adults fouled on watercrafts and/or aquaculture facilities/organisms.

Contrasting with high genetic exchange likely associated with human activities at both fine and large geographic scales, several neighboring populations exhibited significant genetic differentiation (Table 2; Figs. 5 and 6). Because most of the populations analyzed here were sampled from semienclosed or enclosed waters (Table 1), the high genetic differentiation between neighboring populations likely resulted from restricted gene flow associated with the configuration of these sites. *Ciona* larvae may be unable to disperse naturally outside of semienclosed or enclosed waters. A similar genetic pattern has been observed in other invasive ascidians, including *Styela clava* (Dupont et al. 2009).

Several populations exhibited lower genetic diversity and higher genetic differentiation in both species. For spA on the west coast, population MO (Monterey Bay), sampled from open sea shore, had lower genetic diversity than other populations (Table 1; Table S1). Compared to other sites such as ports and marinas with a large number of pathways available, a slower natural dispersal with limited numbers of propagules can result in founder effects, leading to low genetic diversity (Table 1) and high interpopulation genetic differentiation (Table 2). For spB on the east coast, all tests based on microsatellites including pairwise *F*_ST_, Bayesian clustering, and 3D-FCA illustrated that four populations (MA, MB, ST, LU) were divergent from all other populations (Table 1; Figs. 5 and 6). Analyses of microsatellite allelic variation indicated significantly lower genetic diversity in these populations (Table S2). The observed lower level of genetic diversity and significant differentiation can result from three possible processes: separate introductions from genetically distinct sources, rapid selection due to local adaptation, and bottlenecks during secondary range expansion. Multiple lines of evidence suggest that the first two processes are unlikely to be major determinants. Detailed analysis of microsatellite alleles did not show many unique alleles, with only 19 (out of 239, 7.9%) in these four populations. Regionally, given the intensive sampling and relatively low genetic differentiation of the remaining populations on the east coast (Table 2B; Figs. 5B and 6B), a genetically distinct source does not likely exist on the east coast. Globally, genetic analyses revealed a much lower level of genetic differentiation (Zhan et al. 2010). Bayesian clustering analysis based on a combined dataset obtained here and from Zhan et al. (2010) still separated these four populations (Fig. S2). Consequently, introductions from genetically distinct sources both regionally and globally were unlikely. The lower genetic diversity at multiple microsatellite loci (Table S1) suggests that direct selection and/or genetic hitchhiking are likely not responsible for the observed pattern. By contrast to the lower genetic diversity in these four populations, one neighboring population CT (Chester marina) exhibited a level of genetic diversity similar to other populations on the east coast (Fig. 2; Table 1). Compared to these four sites, the Chester marina has both relatively higher shipping traffic and nearby aquaculture activities, either of which could mediate gene flow. Considering the lighter commercial shipping to these four sites, these populations may have been accidentally seeded by limited numbers of propagules transferred via watercrafts, or by natural dispersal from neighboring sources. Population bottlenecks during secondary range expansions can lead to sharp decrease of genetic diversity (see examples in Roman and Darling 2007), and the loss of allelic diversity can result in a high level of genetic differentiation (see e.g., Zhan et al. 2009).

### Inconsistent patterns based on different markers

Interestingly, we observed inconsistent patterns of genetic diversity when using mtDNA and nuclear microsatellites in both species (Table 1; Table S1). The two newly colonized populations of spB, BR (Brudenell River) and MR (Murray River), showed relatively low mtDNA diversity (*n* = 3, *h* = 0.191), though the pattern was not repeated with microsatellite loci (Table 1). In addition, the two populations, MA (Martin's River) and ST (Stone Hurst) that possibly experienced a genetic bottleneck had lower microsatellite diversity but a similar level of mtDNA diversity to other populations (Table 1; Fig. 2). A similar pattern was also detected in population PH (Port Hueneme) of spA (Table 1). Given the difference of effective population size between mtDNA and nuclear markers, genetic drift associated with population size decrease during postestablishment spread may be responsible for producing this inconsistency. In addition, different selection pressures and/or genetic hitchhiking events on mitochondrial versus nuclear genomes could also lead to this discordance.

## Conclusions

Our genetic surveys add to growing evidence that complex genetic patterns can emerge in invasive species, including closely related species having similar biological characteristics. In addition, inconsistent genetic patterns also can be detected when using two different types of markers, that is, mtDNA versus nuclear microsatellites. Detailed analyses suggest that multiple factors, including random genetic drift associated with population demography change, rapid selection due to strong local adaptation, varying propensity for human-mediated propagule dispersal, as well as their interactions, could be responsible for such genetic complexities. In addition, in order to avoid possible biased results caused by one type of molecular marker, our study highlights the necessity of use of both mtDNA and nuclear markers to perform genetic analysis in invasive species. The complex genetic patterns observed here, coupled with multiple putative factors causing such complexities, open a window for us to deeply investigate mechanisms of rapid evolution associated with fast environmental changes due to frequent anthropogenic activities.

## Supporting Information

**Figure S1.** Bayesian inference (BI) tree (A) and haplotype networks generated with TCS for *Ciona intestinalis* spA (B)and spB (C) based on a combined mitochondrial dataset obtained from the present study and Zhan *et al*. (2010).

**Figure S2.** Bayesian inference (BI) of population structure for *Ciona intestinalis* spA (A) and spB (B) based on a combined dataset obtained from the present study and Zhan*et al*. (2010).

**Table S1.** Genetic diversity at eight microsatellite loci for two highly invasive species, *Ciona intestinalis* spA (west coast) and spB (east coast) collected from North America.

**Table S2.** *P*-values for the exact test of difference in allelic richness (below diagonal) and expected heterozygosity (above diagonal) using nonparameteric statistics (Mann–Whitney *U* test) for the two highly invasive species, *Ciona intestinalis* spA on the west coast (S2-1) and spB on the east coast (S2-2) of North America.
