# Supplementary material for: Complex genetic patterns in closely related colonizing invasive species
Source: Ecol Evol. 2012 Jul;2(7):1331–46. doi: 10.1002/ece3.258 (PMC3434944; doi:10.1002/ece3.258)
Supplement: Supplementary file 1 [file ece30002-1331-SD1.pdf]

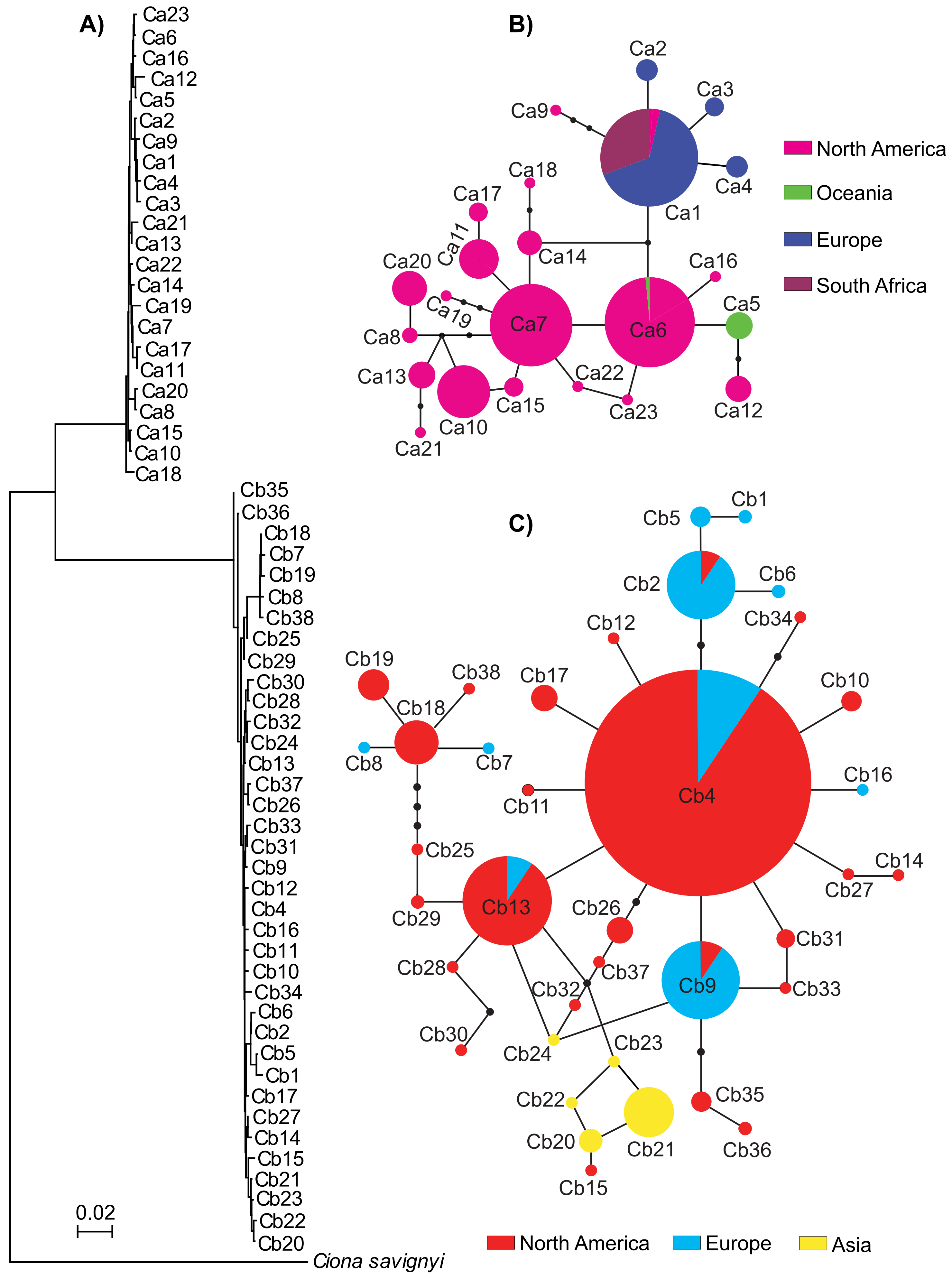

A)
Ca23
Ca6
Ca16
Ca12
Ca5
Ca2
Ca9
Ca1
Ca4
Ca3
Ca21
Ca13
Ca22
Ca14
Ca19
Ca7
Ca17
Ca11
Ca20
Ca8
Ca15
Ca10
Ca18
Cb35
Cb36
Cb18
Cb7
Cb19
Cb8
Cb38
Cb25
Cb29
Cb30
Cb28
Cb32
Cb24
Cb13
Cb37
Cb26
Cb33
Cb31
Cb9
Cb12
Cb4
Cb16
Cb11
Cb10
Cb34
Cb6
Cb2
Cb5
Cb1
Cb17
Cb27
Cb14
Cb15
Cb21
Cb23
Cb22
Cb20
Ciona savignyi
0.02
B)
North America
Oceania
Europe
South Africa
C)
North America
Europe
Asia
