# Supplementary figures and images for: Complex genetic patterns in closely related colonizing invasive species
Source: Ecol Evol. 2012 Jul;2(7):1331–46. doi: 10.1002/ece3.258 (PMC3434944; doi:10.1002/ece3.258)

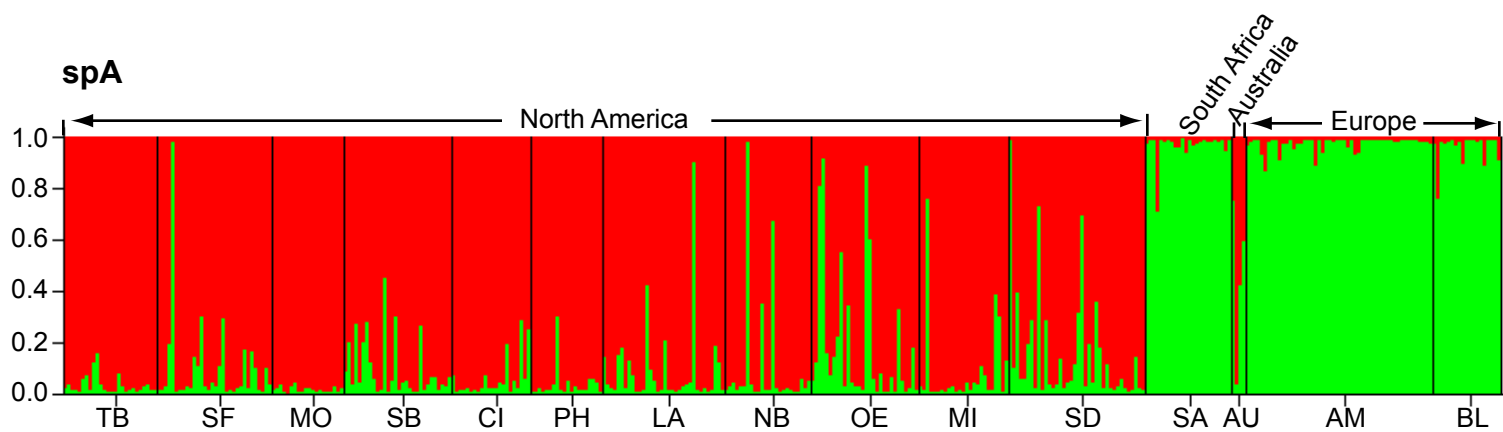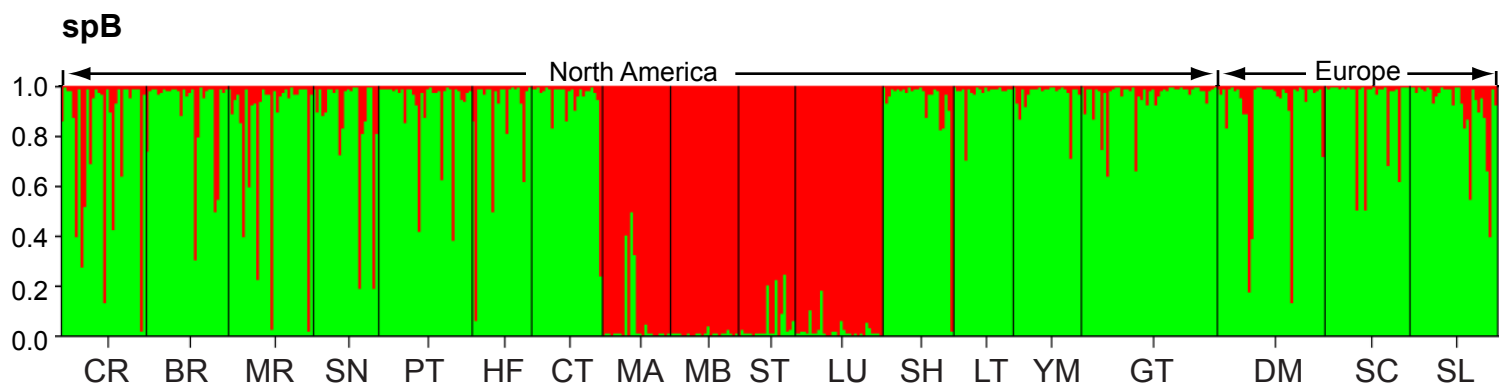

Supplement: Supplementary file 2 [file ece30002-1331-SD2.pdf]
