# Supplementary material for: Complex genetic patterns in closely related colonizing invasive species
Source: Ecol Evol. 2012 Jul;2(7):1331–46. doi: 10.1002/ece3.258 (PMC3434944; doi:10.1002/ece3.258)
Supplement: Supplementary file 3 [file ece30002-1331-SD3.doc]

**Table S1** Genetic diversity at eight microsatellite loci for two highly invasive species, *Ciona intestinalis* spA (west coast) and spB (east coast) collected from North America. *A*, number of alleles; *A*_r_, allele richness; *H*_O_, observed heterozygosity; *H*_E_, expected heterozygosity; *P*_HW_, exact *P*-value for Hardy-Weinberg equilibrium test. The significance after sequential Bonferroni correction was bolded.

| ID | Index | Microsatellite loci | | | | | | | | | Average |
| --- | --- | --- | --- | --- | --- | --- | --- | --- | --- | --- | --- |
|  |  | Cin-1 | Cin-10 | Cin-12 | Cin-13 | Cin-14 | Cin-15 | Cin-16 | Cin-17 |  | |
| East coast (*Ciona intestinalis* spB) | | | | | | | | | | | |
| CR | *A*/*A*_r_ | 6/4.8 | 8/6.7 | 12/7.8 | 9/8.2 | 5/4.5 | 11/9.4 | 11/8.3 | 13/9.8 | 9.4/7.4 | |
|  | *H*_O_ | 0.154 | 0.269 | 0.724 | 0.136 | 0.105 | 0.087 | 0.586 | 0.333 | 0.299 | |
|  | *H*_E_ | 0.704 | 0.844 | 0.817 | 0.892 | 0.656 | 0.908 | 0.858 | 0.902 | 0.823 | |
|  | *P*_HW_ | **0.0000** | **0.0000** | 0.0620 | **0.0000** | **0.0000** | **0.0000** | **0.0000** | **0.0000** | - | |
| BR | *A*/*A*_r_ | 6/4.6 | 7/6.6 | 9/7.3 | 10/8.7 | 6/5.1 | 10/7.9 | 14/9.1 | 11/8.0 | 9.1/7.2 | |
|  | *H*_O_ | 0.276 | 0.250 | 0.621 | 0.111 | 0.167 | 0.250 | 0.500 | 0.522 | 0.337 | |
|  | *H*_E_ | 0.697 | 0.855 | 0.826 | 0.883 | 0.630 | 0.866 | 0.853 | 0.870 | 0.810 | |
|  | *P*_HW_ | **0.0000** | **0.0000** | **0.0005** | **0.0000** | **0.0000** | **0.0000** | **0.0000** | **0.0008** | - | |
| MR | *A*/*A*_r_ | 6/4.8 | 8/6.4 | 12/7.8 | 9/8.0 | 4/3.7 | 9/8.2 | 11/8.4 | 12/9.2 | 8.9/7.1 | |
|  | *H*_O_ | 0.154 | 0.269 | 0.724 | 0.136 | 0.105 | 0.130 | 0.621 | 0.333 | 0.309 | |
|  | *H*_E_ | 0.704 | 0.758 | 0.817 | 0.888 | 0.622 | 0.889 | 0.862 | 0.897 | 0.805 | |
|  | *P*_HW_ | **0.0000** | **0.0000** | 0.0691 | **0.0000** | **0.0000** | **0.0000** | **0.0002** | **0.0000** | - | |
| SN | *A*/*A*_r_ | 8/6.9 | 7/6.2 | 8/6.7 | 10/8.4 | 9/7.2 | 10/9.1 | 10/8.0 | 6/5.0 | 8.5/7.2 | |
|  | *H*_O_ | 0.182 | 0.0476 | 0.696 | 0.222 | 0.087 | 0.278 | 0.435 | 0.200 | 0.268 | |
|  | *H*_E_ | 0.745 | 0.798 | 0.825 | 0.806 | 0.808 | 0.906 | 0.881 | 0.726 | 0.812 | |
|  | *P*_HW_ | **0.0000** | **0.0000** | **0.0003** | **0.0000** | **0.0000** | **0.0000** | **0.0000** | **0.0000** | - | |
| PT | *A*/*A*_r_ | 8/5.2 | 8/6.6 | 13/8.4 | 10/7.2 | 14/8.8 | 11/7.1 | 13/8.6 | 4/3.6 | 10.1/6.9 | |
|  | *H*_O_ | 0.394 | 0.406 | 0.727 | 0.367 | 0.344 | 0.097 | 0.394 | 0.172 | 0.363 | |
|  | *H*_E_ | 0.638 | 0.815 | 0.857 | 0.808 | 0.851 | 0.720 | 0.875 | 0.607 | 0.771 | |
|  | *P*_HW_ | **0.0000** | **0.0000** | 0.0429 | **0.0000** | **0.0000** | **0.0000** | **0.0000** | **0.0000** | - | |
| HF | *A*/*A*_r_ | 13/9.8 | 9/6.9 | 11/7.6 | 14/10.3 | 10/7.8 | 9/8.0 | 9/7.4 | 6/5.5 | 10.1/7.9 | |
|  | *H*_O_ | 0.263 | 0.368 | 0.667 | 0.389 | 0.158 | 0.056 | 0.500 | 0.111 | 0.314 | |
|  | *H*_E_ | 0.868 | 0.814 | 0.801 | 0.906 | 0.794 | 0.849 | 0.835 | 0.741 | 0.826 | |
|  | *P*_HW_ | **0.0000** | **0.0001** | 0.1394 | **0.0000** | **0.0000** | **0.0000** | **0.0006** | **0.0000** | - | |
| CT | *A*/*A*_r_ | 10/6.3 | 12/8.9 | 13/9.2 | 11/8.8 | 11/8.3 | 12/8.7 | 11/9.0 | 9/6.4 | 11.1/8.2 | |
|  | *H*_O_ | 0.240 | 0.292 | 0.640 | 0.227 | 0.435 | 0.261 | 0.542 | 0.217 | 0.357 | |
|  | *H*_E_ | 0.742 | 0.878 | 0.881 | 0.889 | 0.852 | 0.808 | 0.898 | 0.710 | 0.832 | |
|  | *P*_HW_ | **0.0000** | **0.0000** | **0.0021** | **0.0000** | **0.0000** | **0.0000** | **0.0000** | **0.0000** | - | |
| MA | *A*/*A*_r_ | 2/2.0 | 3/2.8 | 8/5.8 | 4/4.0 | 5/4.3 | 3/3.0 | 3/2.9 | 5/4.6 | 4.1/3.7 | |
|  | *H*_O_ | 0.000 | 0.118 | 0.708 | 0.100 | 0.318 | 0.067 | 0.000 | 0.261 | 0.196 | |
|  | *H*_E_ | 0.193 | 0.314 | 0.762 | 0.489 | 0.582 | 0.577 | 0.543 | 0.619 | 0.510 | |
|  | *P*_HW_ | **0.0022** | 0.0087 | 0.6389 | **0.0005** | **0.0005** | **0.0000** | **0.0000** | **0.0001** | - | |

**Table S1** Continued

| ID | Index | Microsatellite loci | | | | | | | | Average |
| --- | --- | --- | --- | --- | --- | --- | --- | --- | --- | --- |
|  |  | Cin-1 | Cin-10 | Cin-12 | Cin-13 | Cin-14 | Cin-15 | Cin-16 | Cin-17 |  |
| MB | *A*/*A*_r_ | 6/4.0 | 5/4.1 | 8/6.6 | 3/2.9 | 5/4.3 | 5/4.0 | 6/5.1 | 5/4.5 | 5.4/4.3 |
|  | *H*_O_ | 0.130 | 0.217 | 0.625 | 0.063 | 0.375 | 0.150 | 0.045 | 0.333 | 0.242 |
|  | *H*_E_ | 0.506 | 0.640 | 0.799 | 0.575 | 0.556 | 0.458 | 0.642 | 0.714 | 0.611 |
|  | *P*_HW_ | **0.0000** | **0.0001** | 0.0172 | **0.0000** | **0.0018** | **0.0001** | **0.0000** | **0.0000** | - |
| ST | *A*/*A*_r_ | 4/3.5 | 7/5.7 | 8/6.8 | 4/4.0 | 8/5.9 | 6/4.9 | 7/6.5 | 3/2.5 | 5.9/5.0 |
|  | *H*_O_ | 0.150 | 0.100 | 0.700 | 0.200 | 0.250 | 0.111 | 0.000 | 0.100 | 0.201 |
|  | *H*_E_ | 0.637 | 0.687 | 0.776 | 0.626 | 0.621 | 0.727 | 0.815 | 0.337 | 0.653 |
|  | *P*_HW_ | **0.0000** | **0.0000** | 0.6594 | **0.0008** | **0.0000** | **0.0000** | **0.0000** | **0.0023** | - |
| LU | *A*/*A*_r_ | 5/4.1 | 6/5.3 | 9/7.1 | 4/3.5 | 8/5.7 | 7/5.3 | 8/6.7 | 7/4.7 | 6.8/5.3 |
|  | *H*_O_ | 0.226 | 0.036 | 0.839 | 0.087 | 0.129 | 0.138 | 0.100 | 0.333 | 0.236 |
|  | *H*_E_ | 0.703 | 0.668 | 0.828 | 0.523 | 0.623 | 0.556 | 0.814 | 0.676 | 0.674 |
|  | *P*_HW_ | **0.0000** | **0.0000** | 0.1177 | **0.0000** | **0.0000** | **0.0000** | **0.0000** | **0.0001** | - |
| SH | *A*/*A*_r_ | 5/4.4 | 6/5.4 | 13/8.6 | 14/10.3 | 12/9.4 | 7/5.8 | 9/6.8 | 3/2.5 | 8.6/6.6 |
|  | *H*_O_ | 0.160 | 0.350 | 0.640 | 0.286 | 0.043 | 0.091 | 0.480 | 0.182 | 0.279 |
|  | *H*_E_ | 0.685 | 0.755 | 0.859 | 0.906 | 0.842 | 0.726 | 0.809 | 0.458 | 0.755 |
|  | *P*_HW_ | **0.0000** | **0.0000** | **0.0014** | **0.0000** | **0.0000** | **0.0000** | **0.0010** | 0.0034 | - |
| LT | *A*/*A*_r_ | 7/6.0 | 8/7.1 | 11/7.6 | 11/8.2 | 8/7.2 | 8/6.0 | 8/6.4 | 8/5.9 | 8.6/6.8 |
|  | *H*_O_ | 0.333 | 0.263 | 0.524 | 0.143 | 0.158 | 0.211 | 0.429 | 0.316 | 0.297 |
|  | *H*_E_ | 0.801 | 0.849 | 0.792 | 0.865 | 0.818 | 0.644 | 0.818 | 0.761 | 0.794 |
|  | *P*_HW_ | **0.0000** | **0.0000** | **0.0024** | **0.0000** | **0.0000** | **0.0000** | **0.0002** | **0.0000** | - |
| YM | *A*/*A*_r_ | 12/9.3 | 10/8.3 | 10/7.6 | 15/10.0 | 12/9.7 | 15/10.9 | 15/9.4 | 12/9.2 | 12.6/9.3 |
|  | *H*_O_ | 0.130 | 0.150 | 0.708 | 0.348 | 0.294 | 0.300 | 0.500 | 0.409 | 0.355 |
|  | *H*_E_ | 0.863 | 0.887 | 0.842 | 0.878 | 0.856 | 0.919 | 0.857 | 0.894 | 0.875 |
|  | *P*_HW_ | **0.0000** | **0.0000** | 0.0308 | **0.0000** | **0.0000** | **0.0000** | **0.0000** | **0.0000** | - |
| GT | *A*/*A*_r_ | 15/8.2 | 15/9.4 | 14/9.3 | 13/8.3 | 20/11.2 | 17/8.5 | 14/8.6 | 12/7.6 | 15.0/8.9 |
|  | *H*_O_ | 0.426 | 0.452 | 0.745 | 0.239 | 0.400 | 0.267 | 0.500 | 0.300 | 0.416 |
|  | *H*_E_ | 0.819 | 0.884 | 0.890 | 0.863 | 0.916 | 0.746 | 0.865 | 0.808 | 0.849 |
|  | *P*_HW_ | **0.0000** | **0.0000** | 0.0158 | **0.0000** | **0.0000** | **0.0000** | 0.0083 | **0.0000** | - |
| West coast (*Ciona intestinalis* spA) | | | | | | | | | | |
| ID | Index | Microsatellite loci | | | | | | | | Average |
|  |  | Cin-1 | Cin-10 | Cin-12 | Cin-13 | Cin-14 | Cin-15 | Cin-16 | Cin-17 |  |
| TB | *A*/*A*_r_ | **2/1.4** | **11/10.3** | 2/1.5 | **6/4.9** | **6/5.5** | 11/7.6 | **6/5.1** | **8/6.1** | 6.5/5.3 |
|  | *H*_O_ | 0.040 | 0.385 | 0.042 | 0.381 | 0.333 | 0.400 | 0.100 | 0.739 | 0.302 |
|  | *H*_E_ | 0.040 | 0.914 | 0.042 | 0.511 | 0.765 | 0.756 | 0.738 | 0.752 | 0.565 |
|  | *P*_HW_ | - | **0.0000** | - | 0.0551 | **0.0000** | **0.0000** | **0.0000** | 0.8068 | - |

**Table S1 Continued**

| ID | Index | Microsatellite loci | | | | | | | | Average |
| --- | --- | --- | --- | --- | --- | --- | --- | --- | --- | --- |
|  |  | Cin-1 | Cin-1 | Cin-1 | Cin-1 | Cin-1 | Cin-1 | Cin-1 | Cin-1 |  |
| SF | *A*/*A*_r_ | 3/2.0 | 16/11.4 | 3/2.6 | 4/3.9 | 9/7.3 | 12/8.9 | 6/5.1 | 6/4.9 | 7.4/5.8 |
|  | *H*_O_ | 0.033 | 0.591 | 0.107 | 0.129 | 0.154 | 0.200 | 0.000 | 0.633 | 0.231 |
|  | *H*_E_ | 0.098 | 0.904 | 0.201 | 0.514 | 0.833 | 0.869 | 0.731 | 0.742 | 0.611 |
|  | *P*_HW_ | 0.0180 | **0.0009** | 0.0082 | **0.0000** | **0.0000** | **0.0000** | **0.0000** | 0.3024 | - |
| MO | *A*/*A*_r_ | 2/2.0 | 3/3.0 | 3/2.2 | 4/3.8 | 3/3.0 | 5/4.9 | 4/3.3 | 5/4.6 | 3.6/3.4 |
|  | *H*_O_ | 0.158 | 0.091 | 0.056 | 0.000 | 0.067 | 0.063 | 0.059 | 0.500 | 0.124 |
|  | *H*_E_ | 0.235 | 0.567 | 0.110 | 0.597 | 0.646 | 0.784 | 0.316 | 0.681 | 0.492 |
|  | *P*_HW_ | 0.2581 | **0.0009** | 0.0297 | **0.0000** | **0.0000** | **0.0000** | **0.0001** | 0.0047 | - |
| SB | *A*/*A*_r_ | **4/2.8** | **21/13.9** | 9/6.0 | 2/2.0 | **8/5.9** | **15/9.8** | **5/4.1** | 6/5.1 | 8.8/6.2 |
|  | *H*_O_ | 0.179 | 0.522 | 0.333 | 0.143 | 0.483 | 0.643 | 0.222 | 0.667 | 0.399 |
|  | *H*_E_ | 0.202 | 0.927 | 0.718 | 0.195 | 0.748 | 0.860 | 0.560 | 0.746 | 0.619 |
|  | *P*_HW_ | 0.0639 | **0.0000** | **0.0000** | 0.2580 | 0.0047 | 0.0336 | **0.0002** | 0.4345 | - |
| CI | *A*/*A*_r_ | **1/1.0** | **6/6.0** | **4/3.4** | 5/4.2 | 3/3.0 | **8/6.9** | **4/4.0** | **4/4.0** | 4.9/4.1 |
|  | *H*_O_ | **-** | 0.273 | 0.143 | 0.182 | 0.067 | 0.412 | 0.083 | 0.611 | 0.253 |
|  | *H*_E_ | **-** | 0.792 | 0.338 | 0.429 | 0.480 | 0.806 | 0.685 | 0.738 | 0.610 |
|  | *P*_HW_ | - | **0.0001** | **0.0000** | **0.0000** | **0.0000** | **0.0000** | **0.0000** | 0.0401 | - |
| PH | *A*/*A*_r_ | **2/1.8** | **5/5.0** | 4/3.5 | 6/5.4 | **6/5.9** | **2/2.0** | **3/3.0** | 5/4.3 | 4.1/3.9 |
|  | *H*_O_ | 0.000 | 0.077 | 0.176 | 0.222 | 0.214 | 0.313 | 0.278 | 0.471 | 0.219 |
|  | *H*_E_ | 0.102 | 0.785 | 0.362 | 0.741 | 0.817 | 0.466 | 0.538 | 0.693 | 0.563 |
|  | *P*_HW_ | 0.0276 | **0.0000** | 0.0059 | **0.0000** | **0.0000** | 0.2722 | 0.0120 | 0.0784 | - |
| LA | *A*/*A*_r_ | **5/2.3** | **11/9.9** | 5/3.2 | 5/3.8 | **8/6.4** | **9/7.3** | **5/4.5** | 8/6.2 | 7.0/5.5 |
|  | *H*_O_ | 0.091 | 0.333 | 0.176 | 0.103 | 0.269 | 0.333 | 0.167 | 0.483 | 0.245 |
|  | *H*_E_ | 0.118 | 0.880 | 0.269 | 0.387 | 0.789 | 0.703 | 0.679 | 0.786 | 0.576 |
|  | *P*_HW_ | 0.0967 | **0.0000** | **0.0004** | **0.0000** | **0.0000** | **0.0000** | **0.0000** | 0.0022 | - |
| NB | *A*/*A*_r_ | **1/1.0** | 13/11.7 | 4/3.2 | 5/4.5 | 4/4.0 | 9/7.8 | 6/4.7 | 5/4.4 | 6.6/5.2 |
|  | *H*_O_ | **-** | 0.250 | 0.130 | 0.474 | 0.083 | 0.450 | 0.158 | 0.625 | 0.310 |
|  | *H*_E_ | **-** | 0.940 | 0.310 | 0.680 | 0.707 | 0.850 | 0.710 | 0.717 | 0.702 |
|  | *P*_HW_ | - | **0.0000** | **0.0001** | 0.1976 | **0.0000** | **0.0008** | **0.0000** | 0.1592 | - |
| OE | *A*/*A*_r_ | 4/3.2 | 14/10.2 | 7/6.2 | 4/3.6 | 9/6.9 | 15/10.5 | 7/6.3 | 8/6.3 | 8.5/6.7 |
|  | *H*_O_ | 0.038 | 0.273 | 0.429 | 0.233 | 0.565 | 0.538 | 0.300 | 0.643 | 0.377 |
|  | *H*_E_ | 0.250 | 0.845 | 0.655 | 0.656 | 0.801 | 0.902 | 0.726 | 0.794 | 0.703 |
|  | *P*_HW_ | **0.0000** | **0.0000** | **0.0010** | **0.0000** | 0.0255 | **0.0000** | **0.0001** | 0.5200 | - |
| MI | *A*/*A*_r_ | **1/1.0** | 13/10.6 | 4/3.2 | 4/3.1 | 9/8.1 | 11/9.5 | 6/5.6 | 5/4.5 | 7.4/5.7 |
|  | *H*_O_ | **-** | 0.588 | 0.160 | 0.160 | 0.333 | 0.471 | 0.067 | 0.636 | 0.345 |
|  | *H*_E_ | **-** | 0.897 | 0.290 | 0.440 | 0.817 | 0.886 | 0.779 | 0.728 | 0.691 |
|  | *P*_HW_ | **-** | **0.0000** | 0.0030 | **0.0002** | **0.0000** | **0.0000** | **0.0000** | 0.6299 | - |

**Table S1** Continued

| ID | Index | Microsatellite loci | | | | | | | | Average |
| --- | --- | --- | --- | --- | --- | --- | --- | --- | --- | --- |
|  |  | Cin-1 | Cin-10 | Cin-12 | Cin-18 | Cin-14 | Cin-15 | Cin-16 | Cin-17 |  |
| SD | *A*/*A*_r_ | 3/2.5 | 19/12.5 | 6/5.3 | 6/4.3 | 11/7.4 | 22/13.3 | 8/5.5 | 8/4.9 | 10.4/7.0 |
|  | *H*_O_ | 0.083 | 0.214 | 0.588 | 0.086 | 0.750 | 0.750 | 0.167 | 0.579 | 0.397 |
|  | *H*_E_ | 0.227 | 0.908 | 0.770 | 0.416 | 0.806 | 0.939 | 0.728 | 0.694 | 0.692 |
|  | *P*_HW_ | **0.0007** | **0.0000** | **0.0007** | **0.0000** | 0.0137 | **0.0000** | **0.0000** | 0.0489 | - |
