# Supplementary material for: Complex genetic patterns in closely related colonizing invasive species
Source: Ecol Evol. 2012 Jul;2(7):1331–46. doi: 10.1002/ece3.258 (PMC3434944; doi:10.1002/ece3.258)
Supplement: Supplementary file 4 [file ece30002-1331-SD4.doc]

**Table S2** *P*-values for the exact test of difference in allelic richness (below diagonal) and expected heterozygosity (above diagonal) using nonparameteric statistics (Mann-Whitney *U* test) for the two highly invasive species, *Ciona intestinalis* spA on the west coast (Table S2-1) and spB on the east coast (Table S2-2) of North America. The population names as per Table 1.

| **S2-1** | CR | BR | MR | SN | PT | HF | CT | MA | MB | ST | LU | SH | LT | YM | GT |
| --- | --- | --- | --- | --- | --- | --- | --- | --- | --- | --- | --- | --- | --- | --- | --- |
| CR | **** | 0.600 | 0.598 | 0.462 | 0.172 | 0.600 | 0.916 | **0.002** | **0.003** | **0.009** | **0.012** | 0.294 | 0.248 | 0.401 | 0.753 |
| BR | 0.674 | **** | 0.753 | 0.600 | 0.294 | 0.674 | 0.462 | **0.002** | **0.006** | **0.012** | **0.016** | 0.345 | 0.172 | 0.074 | 0.401 |
| MR | 0.674 | 0.999 | **** | 0.834 | 0.294 | 1.000 | 0.674 | **0.002** | **0.009** | **0.027** | **0.027** | 0.401 | 0.600 | 0.294 | 0.462 |
| SN | 0.315 | 0.958 | 0.916 | **** | 0.674 | 0.636 | 0.563 | **0.002** | **0.002** | **0.012** | **0.027** | 0.600 | 0.753 | **0.036** | 0.141 |
| PT | 0.420 | 0.833 | 0.916 | 0.916 | **** | 0.529 | 0.141 | **0.003** | **0.016** | 0.074 | 0.093 | 1.000 | 0.916 | **0.009** | 0.066 |
| HF | 0.834 | 0.529 | 0.674 | 0.345 | 0.345 | **** | 0.529 | **0.001** | **0.002** | **0.005** | **0.012** | 0.294 | 0.431 | **0.036** | 0.294 |
| CT | 0.401 | 0.141 | 0.172 | 0.141 | 0.093 | 0.529 | **** | **0.002** | **0.002** | **0.006** | **0.006** | 0.248 | 0.172 | 0.345 | 0.600 |
| MA | **0.002** | **0.002** | **0.005** | **0.001** | **0.005** | **0.001** | **0.001** | **** | 0.294 | **0.027** | **0.046** | **0.016** | **0.002** | **0.001** | **0.001** |
| MB | **0.005** | **0.003** | **0.021** | **0.002** | **0.012** | **0.001** | **0.002** | 0.294 | **** | 0.401 | 0.248 | **0.027** | **0.003** | **0.001** | **0.001** |
| ST | **0.027** | **0.021** | **0.046** | **0.009** | **0.027** | **0.003** | **0.003** | 0.103 | 0.529 | **** | 1.000 | 0.115 | **0.012** | **0.001** | **0.002** |
| LU | **0.046** | **0.046** | 0.059 | **0.021** | 0.074 | **0.003** | **0.003** | **0.027** | 0.093 | 0.753 | **** | 0.115 | **0.046** | **0.001** | **0.005** |
| SH | 0.600 | 0.753 | 0.916 | 0.674 | 0.916 | 0.294 | 0.248 | **0.024** | **0.046** | 0.208 | 0.208 | **** | 0.674 | **0.021** | 0.093 |
| LT | 0.294 | 0.401 | 0.345 | 0.401 | 0.529 | 0.115 | **0.016** | **0.001** | **0.003** | **0.016** | **0.012** | 0.674 | **** | **0.005** | 0.066 |
| YM | 0.059 | **0.006** | **0.012** | **0.005** | **0.006** | 0.093 | **0.027** | **0.001** | **0.001** | **0.001** | **0.001** | **0.046** | **0.001** | **** | 0.529 |
| GT | 0.172 | **0.036** | **0.036** | **0.016** | **0.036** | 0.115 | 0.674 | **0.001** | **0.001** | **0.001** | **0.001** | **0.015** | **0.002** | 0.294 | **** |

| **S2-2** | TB | SF | MO | SB | CI | PH | LA | NB | OE | MI | SD |
| --- | --- | --- | --- | --- | --- | --- | --- | --- | --- | --- | --- |
| TB | **** | 0.753 | 0.462 | 0.916 | 0.862 | 0.834 | 0.916 | 0.908 | 0.462 | 0.418 | 0.462 |
| SF | 0.916 | **** | 0.294 | 0.916 | 0.563 | 0.462 | 0.674 | 1.000 | 0.834 | 0.728 | 0.674 |
| MO | 0.103 | 0.092 | **** | 0.294 | 0.203 | 0.462 | 0.248 | 0.050 | **0.027** | 0.082 | 0.074 |
| SB | 0.793 | 0.752 | 0.083 | **** | 0.728 | 0.462 | 0.753 | 1.000 | 0.600 | 0.487 | 0.529 |
| CI | 0.278 | 0.344 | 0.292 | 0.318 | **** | 0.908 | 0.728 | 0.565 | 0.355 | 0.338 | 0.452 |
| PH | 0.294 | 0.270 | 0.493 | 0.172 | 0.875 | **** | 0.753 | 0.355 | 0.208 | 0.271 | 0.294 |
| LA | 0.834 | 0.875 | **0.013** | 0.753 | 0.115 | 0.066 | **** | 0.355 | 0.345 | 0.247 | 0.345 |
| NB | 0.462 | 0.674 | 0.172 | 0.529 | 0.370 | 0.529 | 0.462 | **** | 1.000 | 0.655 | 0.817 |
| OE | 0.278 | 0.528 | **0.008** | 0.293 | 0.065 | **0.016** | 0.528 | 0.318 | **** | 0.817 | 0.834 |
| MI | 0.916 | 1.000 | 0.141 | 0.834 | 0.371 | 0.294 | 0.752 | 0.792 | 0.430 | **** | 0.862 |
| SD | 0.600 | 0.401 | **0.013** | 0.753 | 0.074 | 0.083 | 0.674 | 0.208 | 0.834 | 0.600 | **** |
